# Association between health literacy and medical care costs in an integrated healthcare system: a regional population based study

**DOI:** 10.1186/s12913-015-0887-z

**Published:** 2015-06-27

**Authors:** Jolie N. Haun, Nitin R. Patel, Dustin D. French, Robert R. Campbell, Douglas D. Bradham, William A. Lapcevic

**Affiliations:** HSR&D Center of Innovation on Disability and Rehabilitation Research, James A. Haley Veterans Hospital, 8900 Grand Oak Circle (151R), Tampa, FL 33637 USA; Department of Community & Family Health, University of South Florida College of Public Health, 13201 Bruce B. Downs Blvd. (MDC 56), Tampa, FL 33612-3805 USA; Department of Ophthalmology and Center for Healthcare Studies and VA Center of Excellence, Northwestern University Feinberg School of Medicine, 645 N. Michigan Ave. Suite 440, Chicago, IL 60611 USA; Robert J Dole VAMC, 5500 East Kellogg Ave., Wichita, KS 67218 USA; American Institutes for Research, Health and Social Development Program, Health Policy and Research Group, 1000 Thomas Jefferson Street, NW, Washington, DC 20007-3835 USA

**Keywords:** Retrospective, Health care costs, VA health care system

## Abstract

**Background:**

Low health literacy is associated with higher health care utilization and costs; however, no large-scale studies have demonstrated this in the Veterans Health Administration (VHA). This research evaluated the association between veterans’ health literacy and their subsequent VHA health care costs across a three-year period.

**Methods:**

This retrospective study used a Generalized Linear Model to estimate the relative association between a patient’s health literacy and VHA medical costs, adjusting for covariates. Secondary data sources included electronic health records and administrative data in the VHA (e.g., Medical and DCG SAS Datasets and DSS-National Data Extracts). Health literacy assessments and identifiers were electronically retrieved from the originating health system. Demographic and cost data were retrieved from the VHA centralized databases for the corresponding patients who had VHA use in all three years.

**Results:**

In a study of 92,749 veterans with service utilization from 2007–2009, average per patient cost for those with inadequate and marginal health literacy was significantly higher ($31,581 [95 % CI: $30,186 - $32,975]; $23,508 [95 % CI: $22,749 - $24,268]) than adequate health literacy ($17,033 [95 % CI: $16,810 - $17,255]). Estimated three-year cost associated with veterans’ with marginal and inadequate health literacy was $143 million dollars more than those with adequate health literacy.

**Conclusions:**

Analyses suggest when controlling for other person-level factors within the VHA integrated healthcare system, lower health literacy is a significant independent factor associated with increased health care utilization and costs. This study confirms the association of lower health literacy with higher medical service utilization and pharmacy costs for veterans enrolled in the VHA. Confirmation of higher costs of care associated with lower health literacy suggests that interventions might be designed to remediate health literacy needs and reduce expenditures. These analyses suggest 17.2 % (inadequate & marginal) of the Veterans in this population account for almost one-quarter (24 %) of VA medical and pharmacy cost for this 3-year period. Meeting the needs of those with marginal and inadequate health literacy could produce potential economic savings of approximately 8 % of total costs for this population.

## Background

Health literacy, though an evolving concept [1], is defined as the, “degree to which individuals have the capacity to obtain, process, and understand basic health information and services needed to make appropriate health decisions” [2] and “the capacity of individuals to obtain, interpret, and understand basic health information and services and the competence to use such information and services in ways which enhance health” [3]. Adequate health literacy requires the application of listening, analyzing, and decision-making skills across a variety of health situations such as understanding medication instructions, actively participating in health encounters, and giving consent. Health literacy is an individual determinant [4] that has been associated with utilization of medical services and health care costs. In the general population, inadequate health literacy has been associated with an increased need for disease management, higher medical service utilization among older, racially ethnic minorities, and with low educational attainment [5–9]. In managed care enrollees, individuals with low health literacy have higher medical costs and are less efficient when using services (e.g., more ER visits) than those individuals with adequate health literacy [10]. Some have estimated the costs associated with inadequate health literacy among adults at the national level to be $73 billion annually [11].

With the emerging legislation such as the Veterans Access, Choice, and Accountability Act (VACAA) and the Affordable Care Act (ACA) focused on access to care for Veterans and the nation’s general population, understanding the role of health literacy on utilization and health care cost is imperative. To date, there are few large scale studies published in the literature [10, 11] and none have been conducted among veterans enrolled in the Veterans Health Administration (VHA). The VHA is one of the largest horizontally and vertically integrated health care systems in the United States, with an established national electronic medical record system. As such, this system offers a unique opportunity to evaluate the impacts of health literacy on VHA service utilization and costs for Veterans with varying levels of health literacy. Yet, before launching new interventions to improve health literacy, the economic impact of the problem should be documented within this healthcare system.

This investigation evaluated the association between VHA healthcare cost estimates and lack of adequate health literacy in a large veteran patient population over a 3-year period (2007–2009), accounting for personal and health characteristics. The authors hypothesized that despite the integrated nature of the VHA system, healthcare costs are significantly indirectly and inversely associated with health literacy, such that veterans with lower health literacy have higher healthcare costs across fiscal years. The theoretical basis for selecting the factors to be evaluated in this analysis is supported by Paasche-Orlow and Wolfs proposed causal pathway model between health literacy and health outcomes [12].

## Methods

### Design

This retrospective study used a Generalized Linear Model to estimate the association between a patient’s health literacy and VHA medical costs, adjusting for covariates in a regional population of 112,417 veterans within the VHA. Because this methodology is capturing health literacy at the population level, there is no sample selection bias, thus we are isolating the effect of health literacy without introducing pre-selection bias. As such, we selected a controlled regression analysis to assess the cost profile with controlled variables of interest.

### Population description

A total of 112,417 veterans with a health literacy screening between 2007 and 2009 were identified for the study in the North Florida/South Georgia region. This regional study population is representative of the southeast regions of VHA including both rural and non-rural dwelling veterans, with similar age and gender characteristics of not only VISN 8 but the national VHA patient population, being predominantly older white males. To investigate the impact of health literacy on VHA utilization (i.e., composed of: inpatient, outpatient and/or pharmacy), we analyzed data for patients who had utilization of services in each of the three years, for a final study population of 92,749.

### Data sources

Veterans within the North Florida/South Georgia sub-region of the VHA are screened for health literacy once every five years (unless re-assessment is needed due to trauma or cognitive decline). Electronic health records within the North Florida/South Georgia sub-region of the VHA (VISN 8 region) were accessed to leverage this regional population of veterans that were routinely screened for health literacy between fiscal years 2007 to 2009. This sub-region is unique in that it is an early adopter for routinely assessing and documenting health literacy for their veteran population. The health literacy screening is conducted along with routine “clinical reminders” by clinicians and/or clinical staff during routine clinical care visits. The health literacy screening instrument is composed of a four item tool known as the *BRIEF health literacy screening tool*: (1) How often do you have someone help you read hospital materials? (2) How confident are you filling out medical forms by yourself? (3) How often do you have problems learning about your medical condition because of difficulty understanding written information? and (4) How often do you have a problem understanding what is told to you about your medical condition? These items have been found to effectively identify individuals with inadequate/marginal health literacy skills [13]. Response options are scored on five-point Likert-type scales for each of the items [items 1, 3 & 4 (1 = always to 5 = never); and item 2 (1 = not at all to 5 = extremely)]. The potential summative score from the BRIEF ranges from 4–20 with scores categorized as: (1) inadequate (4–12); (2) marginal (13–16); and adequate (17–20).

Data on workload was extracted from the centralized national Medical SAS datasets and cost data was extracted from the Decision Support System National Data Extracts (NDE). The national Diagnostic Cost Group (DCG) SAS datasets provided the DCG information. The computerized patient records system and other administrative systems store data collected during patient care encounters. These data are assembled at national levels into the VHA Medical SAS (MedSAS) Datasets, [14] Decision Support System - National Data Extracts (DSS-NDE) [15] and Diagnostic Cost Group (DCG) SAS datasets [16], stored on the Austin Information Technology Center mainframe. These files provided expenses, encounters and diagnoses for the study’s subset of screened patients, who also had used VHA care – outpatient, inpatient or pharmacy services – for fiscal years 2007 through 2009.

### Procedure

Health literacy assessment data and corresponding identifiers for the study were electronically retrieved from the originating site and transferred via an intra-agency data transfer agreement to the investigators. Identifiers were then used to access and extract demographic, health condition and cost data from other VHA centralized administrative data sources. These identifiable data were extracted and collected with University of South Florida Institutional Review Board approval (CR6_Pro00000120). Data extracts were cleaned, and checked for administrative data entry errors by the study data managers. The patient identifiers were replaced using a de-identification cross-walk to a unique study ID and then all data were imported into a single analytical file for analyses.

### The main effect (independent) variable is health literacy group

The primary outcome (dependent variable) was each patient’s annual aggregate VHA medical, fee-basis and pharmacy cost for fiscal year (FY) 2007 through 2009. VA inpatient, outpatient and pharmacy cost values from the national DSS-NDE were summed for each patient, in each year. It should be noted that dollar values are nominal, (not adjusted for inflation), and are taken from this VHA’s internal cost allocation system, which are more meaningful to VHA policymakers and administrators than externally valid expense values. Since we seek only to detect the relative within-VHA difference between health literacy groups, these values are sufficient, and do not require further adjustment for inflation or wage differences between facilities. Such adjustments would be necessary if we were comparing outcomes across years or facilities, respectively.

In the VA, cost estimates are recorded for each outpatient unit of care, (i.e., physician, practitioner, labs, and imaging) delivered in both Department of Veterans’ Affairs (VA) Medical Centers and in VA Community-based Outpatient Centers. Expenses for all inpatient care (e.g., acute, extended, observation and non-VA or contractual care) are also recorded. Pharmacy cost data for both outpatient or inpatient care are separately included. Thus, costs for each healthcare encounter (e.g. hospital discharge, specialty service, or outpatient visit) are accounted for in the VHA’s national MedSAS and DSS-NDE datasets for each patient across the national delivery system. Consequently, when aggregated to annual per patient levels, the total resources consumed for that individual whether in local or distant sites (e.g., while vacationing) is captured. We analyzed and report on the annual aggregate of these comprehensive dollar values labeling them as “medical and pharmacy” values to be clear.

### Covariates

Guided by Paasche-Orlow and Wolfs causal model [12], demographics and constructed covariates were extracted for each member of this dataset from national VHA data systems. Diagnostic and procedural codes were processed to provide a risk-adjustment in each year at the patient level, i.e., DCG Hierarchical Condition Category (HCC). The DCG HCC score identifies the relative cost categorization by health status, calculated by accessing the clinical complexity and use of care. This analysis approach serves as a patient level measure of medical co-morbidities [17]. HCC categories were grouped into 5 major chronic disease groups for risk adjustment: Cerebral (HCC 097–099), Heart (HCC 083–084), Vascular (HCC 104–105), Cancer (HCC 007–010), Diabetes (HCC 015–020) and Arrhythmias (HCC 092–093). These conditions are common among veterans and are associated with higher costs of care [18–21]. A patient might be found in multiple conditions, if more than one was documented.

To capture relative levels of priority to access VA care for each veteran, Service Priority levels of 1–8 are routinely assigned to all VHA enrollees generally based on their service-connected disability and income levels. The priority level identifies the amount of copayment for VA services and the relative priority to be seen for non-service connected conditions. We created 4 groups for our analysis: priority 1 and 4 (“catastrophically disabled”), priority 2, 3, and 6 (“moderate disability”), priority 5 (“Medicaid assistance/low income”), and priority 7 and 8 (“no service-connected disability”), which served as the reference group. As a composite measure of access and income, this metric is well understood within the VHA system.

### Multiple imputations

A multiple imputation method was used to replace (12-15 %) missing race and ethnicity in the analytical data. The two step process included creating 5 imputed datasets using the Markov Chain Monte Carlo method to make the data monotone in nature; and then using the monotone regression approach to impute the remaining missing variables [22]. There were no significant differences in the results from the imputed data and the original data.

### Analysis

Patient characteristics and VA medical and pharmacy costs are reported with descriptive statistics. Analyses of bivariate categorical and continuous data were tested with chi-square or Kruskal-Wallis Test. Generalized Linear Model procedures with a log link function and Gamma distribution measured the association of health literacy with annual (2007–2009) patient-level VA medical, fee-basis and pharmacy costs, while other factors are controlled. Generalized Linear Model was used to account for the skewed distribution of the cost data [23]. Guided by the literature, final Generalized Linear Models were adjusted for race, gender, age, marital status, veteran’s service priority level, clinical complexity (co-morbidities and DCG score), and disease categories. We examined the potential for a more complex relationship to exist through Generalized Linear Models that included all covariates and interactions of health literacy with patient demographics and co-morbidity categories, for each year. Coefficients in the interaction models differed only slightly from the more simplified models, which are presented for parsimony [24]. P-values less than 0.05 were considered to be statistically significant. All analyses were conducted with Statistical Analysis Software (SAS, Version 9.3, Cary, NC). Adjusted risk ratios are reported with 95 % confidence intervals (CI).

## Results

We identified 92,749 VA patients with any VA cost data in 2007 through 2009. Health literacy for this population was distributed as: Adequate = 83 %, Marginal = 12 %, Inadequate = 5 %. Three age groups (between 55–84 year olds) represented 75 % of the dataset; therefore, we assigned the oldest sub-group (85 and older) as the reference group because one might expect their expenses to be highest and health literacy lowest, representing 7 % of the study population. The vast majority of the veterans’ were males (94 %), non-minority (White/Caucasian, 85 %) and married (64 %) at the beginning of the study period. About half of the veterans’ (48 %) had a service priority level that represents Medicaid assistant/low income or catastrophically disabled. Population demographics are illustrated in Table 1.

**Table 1:** VA Medical, fee basis and pharmacy average nominal cost per patient, between 2007–2009, by demographic characteristics of patients (n = 92,749)

		Cost from 2007 to 2009
Variables	n (%)	Mean (sd^a^)
Gender*		
Male	87020 (94)	18442 (34316)
Female^b^	5729 (6)	20585 (30278)
Marital Status*		
Divorced	19496 (21)	23801 (40762)
Married^b^	58912 (64)	15857 (29401)
Other	14280 (15)	22540 (39941)
Priority Code*		
Catastrophically disabled	18058 (20)	31315 (46457)
Moderate disability	21013 (23)	14611 (26790)
Medicaid assistance/low income	25864 (28)	22743 (37926)
No service-connected disability^b^	27689 (30)	9399 (19378)
Race^c^*		
Non-minority^b^	67888 (85)	19101 (35206)
Minority	11702 (15)	23074 (37959)
Ethnicity*		
Not Hispanic or Latino^b^	80043 (98)	19080 (34873)
Hispanic or Latino	1695 (2)	19544 (29379)
Age^d^*		
<55	17056 (18)	19813 (35875)
55-64	26106 (28)	22703 (38410)
65-74	22355 (24)	17298 (32646)
75-84	21065 (23)	14643 (28470)
≥85^b^	6167 (7)	15726 (29870)
Annual Per Person VA Expenses by Health Literacy Level* and by Sub-Component		
Total Healthcare Expenses*^e^		
Inadequate	4952 (5)	31581 (50056)
Marginal	10955 (12)	23508 (40550)
Adequate^b^	76842 (83)	17033 (31458)
Inpatient*^e^		
Inadequate	1285 (9)	37878 (61788)
Marginal	2181 (15)	34289 (53340)
Adequate^b^	11050 (76)	26879 (45797)
Outpatient*^e^		
Inadequate	4952 (5)	20748 (23360)
Marginal	10955 (12)	16051 (19947)
Adequate^b^	76842 (83)	12731 (17109)
Fee-Basis*^e^		
Inadequate	1303 (6)	3813 (10934)
Marginal	2706 (13)	2552 (7664)
Adequate^b^	17449 (81)	1922 (7728)
Pharmacy*^e^		
Inadequate	4891 (5)	6071 (18460)
Marginal	10722 (12)	4002 (8213)
Adequate^b^	74922 (83)	3032 (7824)

*Significant differences in cost between categories for all years (Kruskal-Wallis Test) p-value < 0.001

^a^sd = Standard Deviation

^b^Reference Group

^c^Minority = Black/African American, American Indian/Alaska Native, Native Hawaiian/Other Pacific Islander, Asian

^d^Age was documented at time of screening

^e^Population size represents the 2007–2009 combined; numbers vary based on annual utilization

**Fig. 1:**
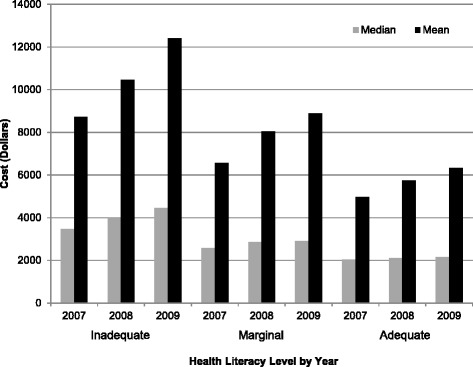
Unadjusted mean and median 2007–2009 VA Medical and Pharmacy Cost by health literacy levels indicate an inverse relationship, with increased cost being associated with lower levels of health literacy. Note: A = Adequate Health Literacy; M = Marginal Health Literacy; I = Inadequate Health Literacy

In Table 1, total overall 3-year costs for VA health care, including inpatient, outpatient, fee-basis, and pharmacy for the study population was $1,722,761,825. Average nominal VA medical, fee-basis and pharmacy cost per patient for the three-years were significantly higher for the lower health literacy levels compare to the adequate group; for example: $17,033 for adequate, $23,508 for marginal and $31,581 for inadequate health literacy. Average nominal cost per patient (2007–2009) for inpatient care increased significantly as health literacy level decreased from adequate, marginal to inadequate ($26,879, $34,289 and $37,878 respectively; p-value < 0.001). Furthermore, outpatient and pharmacy cost for 2007–2009 are significantly higher for veterans’ with inadequate and marginal health literacy, compared to adequate health literacy. Figure 1 shows the unadjusted mean and the median cost by year and by health literacy level. The large difference in the mean and the median values signals the cost data is highly skewed, which is typical.

Chronic condition prevalence’s, derived by HCC disease categories are presented in Table 2 by health literacy level for the three year time period. Prevalence of CHF heart disease among these veterans’ decreased as health literacy level increases from inadequate to adequate (p-value < 0.001). The prevalence of vascular conditions was shown to be significantly lower (104.8 per 1000) among veterans with adequate health literacy, as compared to inadequate health literacy (162.6 per 1000). Overall, prevalence of these chronic conditions (cerebral, vascular, cancer, diabetes and arrhythmias) among these veterans significantly decreased with more adequate health literacy (p-value < 0.001).

**Table 2:** Prevalence (per 1000) of key chronic conditions by health literacy level from 2007 to 2009

	Health literacy level prevalence per 1000		
Conditions (using HCC)	Inadequate	Marginal	Adequate
Cerebral*	73.1	60	46.9
Heart (CHF)*	359.5	334.4	272.2
Vascular*	162.6	140.1	104.8
Cancer*	191	172	148.8
Diabetes*	366.3	344.3	297.9
Arrhythmias*	204.2	182.3	142.6

*Significant differences between Health Literacy and Disease status for all years (Chi-squared Test) p-value < 0.001

### Comparing base and fully interactive models

A base model of independent factors (i.e., gender, marital status, VHA Priority Code, race, ethnicity, age groups, comorbidity score and presence of chronic conditions and health literacy categories) was found to significantly influence both consecutive annual and three-year average estimates of VHA healthcare cost per person. This base model documents the independent, increasing effect of inadequate and marginal health literacy (reference is adequate health literacy) on total annual costs of care in the VHA integrated system, for this population of veterans. Veterans with inadequate health literacy had the greatest relative effect – an increase of 26 %, 32 % and 50 % - on VA medical, fee-basis and pharmacy cost for each year, respectively by year (Table 3, base model), adjusted for included covariates. Overall, the base and interaction Generalized Linear Model showed a significant association between VA medical, fee-basis and pharmacy costs with health literacy, age, gender, marital status, access priority, chronic conditions, and DCG scores (Interaction data not shown).

**Table 3:** Relative cost increase estimates & confidence intervals for factors associated with VA medical, fee basis and pharmacy costs, base and interaction models^a^ (N = 92,749)

Relative Increases in Cost ($) from 2007 to 2009								
	2007		2008		2009		2007-2009	
	Base	Interaction	Base	Interaction	Base	Interaction	Base	Interaction
Variables	Estimate (95 % CI)		Estimate (95 % CI)		Estimate (95 % CI)		Estimate (95 % CI)	
Health Literacy								
Inadequate	1.26** (1.23-1.29)	1.72** (1.45-2.03)	1.32** (1.28-1.36)	1.58** (1.32-1.91)	1.50** (1.45-1.54)	2.32** (1.92-2.8)	1.30** (1.27-1.33)	2.06** (1.76-2.4)
Marginal	1.10** (1.08-1.12)	1.17* (1.04-1.31)	1.14** (1.12-1.17)	1.43** (1.26-1.62)	1.17** (1.15-1.20)	1.83** (1.61-2.07)	1.12** (1.10-1.14)	1.42** (1.28-1.57)
Adequate	(Reference)							
Gender								
Male	0.83** (0.81-0.86)	0.83** (0.81-0.85)	0.83** (0.81-0.85)	0.83** (0.80-0.85)	0.80** (0.78-0.82)	0.81** (0.78-0.83)	0.83** (0.82-0.85)	0.84** (0.82-0.86)
Female	(Reference)							
Marital Status								
Divorced	1.15** (1.14-1.17)	1.16** (1.14-1.18)	1.13** (1.11-1.15)	1.12** (1.10-1.14)	1.18** (1.16-1.19)	1.17** (1.14-1.19)	1.15** (1.14-1.17)	1.15** (1.13-1.16)
Other	1.13** (1.11-1.15)	1.11** (1.09-1.14)	1.13** (1.11-1.15)	1.10** (1.08-1.12)	1.12** (1.10-1.15)	1.13** (1.11-1.16)	1.12** (1.10-1.14)	1.11** (1.09-1.13)
Married	(Reference)							
Priority Code								
Catastrophically disabled	2.11** (2.07-2.14)	2.11** (2.07-2.15)	1.92** (1.89-1.96)	1.90** (1.86-1.94)	1.99** (1.95-2.03)	2.00** (1.96-2.04)	1.88** (1.85-1.91)	1.88** (1.85-1.92)
Medicaid assistance/low income	1.58** (1.56-1.61)	1.59** (1.56-1.61)	1.57** (1.54-1.59)	1.57** (1.54-1.60)	1.60** (1.57-1.63)	1.61** (1.58-1.64)	1.55** (1.53-1.58)	1.56** (1.53-1.58)
Moderate disability	1.25** (1.23-1.27)	1.24** (1.21-1.26)	1.26** (1.24-1.28)	1.25** (1.22-1.27)	1.30** (1.28-1.33)	1.29** (1.26-1.31)	1.26** (1.24-1.28)	1.25** (1.23-1.27)
No service-connected disability	(Reference)							
Race								
Minority	0.98* (0.96-1.00)	0.97* (0.95-0.99)	1.01 (0.99-1.03)	1.00 (0.98-1.03)	1.04* (1.02-1.06)	1.03* (1.01-1.06)	1.01 (1.00-1.03)	1.00 (0.99-1.02)
Non-minority	(Reference)							
Ethnicity								
Hispanic or Latino	1.08* (1.04-1.13)	1.09** (1.04-1.14)	1.03 (0.98-1.08)	1.01 (0.96-1.06)	1.03 (0.99-1.08)	1.02 (0.97-1.08)	1.04* (1.00-1.08)	1.03(0.98-1.07)
Not Hispanic or Latino	(Reference)							
Age								
<55	1.64** (1.59-1.69)	1.68** (1.62-1.73)	1.67** (1.62-1.72)	1.70** (1.64-1.76)	1.56** (1.52-1.61)	1.71** (1.65-1.77)	1.64** (1.60-1.68)	1.71** (1.66-1.76)
55-64	1.62 ** (1.58-1.67)	1.66** (1.61-1.71)	1.65** (1.6-1.69)	1.68** (1.62-1.74)	1.54** (1.50-1.59)	1.69** (1.64-1.75)	1.61** (1.58-1.65)	1.70** (1.65-1.74)
65-74	1.41** (1.37-1.44)	1.43** (1.39-1.47)	1.42** (1.38-1.46)	1.44** (1.39-1.49)	1.26** (1.22-1.29)	1.38** (1.33-1.42)	1.37** (1.34-1.41)	1.44** (1.40-1.48)
75-84	1.11** (1.08-1.14)	1.13** (1.09-1.16)	1.15** (1.12-1.19)	1.18** (1.14-1.22)	1.03* (1.00-1.06)	1.09** (1.06-1.13)	1.10** (1.08-1.13)	1.14** (1.11-1.17)
≥85	(Reference)							
DCG	2.13** (2.11-2.15)	2.22** (2.20-2.25)	2.01** (1.99-2.03)	2.10** (2.07-2.12)	2.01** (1.99-2.03)	2.09** (2.06-2.11)	2.35** (2.33-2.37)	2.47** (2.45-2.50)
Disease Status	(Reference = “no disease”)							
Cerebral	1.23** (1.19-1.28)	1.25** (1.2-1.3)	1.26** (1.21-1.31)	1.27** (1.21-1.33)	1.27** (1.22-1.32)	1.27** (1.21-1.33)	1.14** (1.12-1.17)	1.14** (1.11-1.17)
Heart	1.22** (1.20-1.23)	1.22** (1.20-1.24)	1.29** (1.27-1.31)	1.30** (1.28-1.32)	1.18** (1.16-1.20)	1.19** (1.17-1.21)	1.17** (1.15-1.18)	1.19** (1.17-1.20)
Vascular	1.16** (1.13-1.19)	1.19** (1.16-1.22)	1.24** (1.21-1.27)	1.26** (1.22-1.29)	1.18** (1.15-1.21)	1.19** (1.15-1.22)	1.12** (1.10-1.14)	1.13** (1.11-1.16)
Cancer	1.27** (1.24-1.29)	1.26** (1.24-1.29)	1.32** (1.29-1.34)	1.34** (1.31-1.37)	1.36** (1.34-1.39)	1.36** (1.33-1.39)	1.21** (1.19-1.23)	1.21** (1.19-1.23)
Diabetes	1.25** (1.23-1.26)	1.26** (1.24-1.28)	1.26** (1.24-1.28)	1.28** (1.26-1.30)	1.25** (1.23-1.26)	1.25** (1.23-1.27)	1.21** (1.19-1.22)	1.21** (1.20-1.23)
Arrhythmias	1.14** (1.12-1.17)	1.13** (1.11-1.16)	1.21** (1.19-1.24)	1.23** (1.19-1.26)	1.29** (1.26-1.32)	1.30** (1.27-1.33)	1.14** (1.12-1.15)	1.13** (1.11-1.15)

* = p-value < 0.05, ** = p-value < 0.001

^a^Interaction terms not shown

In 2009, veterans with inadequate health literacy had significantly higher costs than veterans with adequate health literacy after adjusting for other covariates and interactions (RR: 2.32 [95 % CI: 1.92-2.80]) and marginal (RR: 1.83 [95 % CI: 1.61-2.07]). In the same year, veterans with catastrophic disability and Medicaid assistance, or low income, were 100 % and 61 % more costly than veterans with no service-connected disability. In 2009, veterans with cerebral chronic conditions average a 27 % increased expense, with other covariates and interaction controlled, over the veteran who had none of these noted conditions. Those with cancer exhibited a 36 % increase over those who had no chronic conditions. The presence of selected chronic conditions and higher comorbidity scores increased annual costs of care, as expected for all years. Compared to the reference age group (≥85 years old), veterans in other age groups had significantly higher cost.

### Predicted impact of health literacy

The two models (i.e., base and fully interactive) provide insight into the impact on VHA expenditures and the importance of health literacy levels from various common scenarios among veterans (Interactive data not shown). In Table 4, we incrementally changed the risk factors for VHA annual expenses, from the most frequent scenario found in this study population (see Table 1). The multi-factor reference group was: Male veterans aged 55–64 years old, Married, not disabled or Service Connected, non-minority, non-Hispanic, with no Chronic Conditions, and mean comorbidity score. This scenario serves as a reference point against which the other scenarios’ predicted expenditures are compared.

**Table 4:** Three year (2007–2009) predicted costs by risk scenarios

	Health literacy					
	Adequate		Marginal		Inadequate	
	Base Model	Interaction Model	Base Model	Interaction Model	Base Model	Interaction Model
Male aged 55–64 years old, married, not disabled or service connected, non-minority, not Hispanic, with no chronic conditions and mean comorbidity score	$8,342	$8,413	$9,335	$8,957	$10,854	$12,267
% difference between base and interaction model	<1 %		−4 %		13 %	
Male aged 55–64 years old, *divorced*, not disabled or service connected, non-minority, not Hispanic, with no chronic conditions and mean comorbidity score	$9,610	$9,646	$10,754	$10,881	$12,504	$12,746
% difference between base and interaction model	<1 %		1 %		2 %	
Male aged 55–64 years old, *divorced*, *moderately disabled*, non-minority, not Hispanic, with no chronic conditions and mean comorbidity score	$12,117	$12,071	$13,560	$14,395	$15,767	$16,926
% difference between base and interaction model	- < 1 %		6 %		7 %	
Male aged 55–64 years old, *divorced*, *moderately disabled, minority*, not Hispanic, with no chronic conditions and mean comorbidity score	$12,259	$12,113	$13,719	$15,305	$15,952	$17,363
% difference between base and interaction model	- < 1 %		12 %		9 %	
Male aged 55–64 years old, *divorced*, *moderately disabled, minority, Hispanic*, with no chronic conditions and mean comorbidity score	$12,783	$12,434	$14,306	$17,349	$16,633	$18,747
% difference between base and interaction model	−3 %		21 %		13 %	
Male aged 55–64 years old, *divorced*, *moderately disabled, minority, Hispanic*, *heart and diabetes conditions* and mean comorbidity score	$18,019	$17,905	$20,165	$22,800	$23,446	$22,068
% difference between base and interaction model	- < 1 %		13 %		−6 %	
Male aged 55–64 years old, *divorced*, *moderately disabled, minority, Hispanic, heart and diabetes conditions and upper quartile comorbidity score*	$20,312	$20,326	$22,731	$25,367	$26,429	$24,080
% difference between base and interaction model	<1 %		12 %		−9 %	

The three health literacy level columns of Table 4 show: the base and interaction models values, and the percent difference between base and interaction model in the following row. Increased annual expenditures imply potentially more frequent utilization and/or more intense services. Both models consistently predict higher levels of expenditure as health literacy worsens. These increases are not related to increased severity, since the comorbidity scores and chronic conditions control severity, within a particular scenario. An increased healthcare expenditure gradient associated with decreasing health literacy levels is evident. These data suggest that those that are Hispanics with comorbidities and/or the chronic conditions of diabetes and/or heart disease with marginal or inadequate health literacy have higher costs in both the base and interaction variable models.

## Discussion

This study provides a population based large–scale dataset to confirm the long-held belief that there is an inverse association between a veteran’s health literacy and annual VA medical and pharmacy utilization and costs at a population level. Findings suggest after controlling for other well-known demographic and co-morbidity factors that impact costs, health literacy remains a significant independent factor in predicting future health care utilization and costs, consistent with findings of Howard et al. (2005) [10] in a Medicare Managed Care enrollee cohort of 3,260 in 1997. Unexpectedly, this study found that compared to the reference age group of the older old (≥85 years old), younger veterans had significantly higher cost; suggesting that veterans’ VHA annual expenses decrease as they age, even while health literacy, demographics, access to care and complexity of conditions are held constant. Further study is needed to uncover why this might be the case, since our dependent variable included hospital, nursing home and hospice care whether VA-delivered or externally contracted.

This study highlights the importance of health literacy on total costs of care in an integrated medical system, and the value of screening patients for health literacy to demonstrate associated costs; thereby providing quantitative data required of monitoring and remediation. This study is the first published longitudinal insight on costs associated with health literacy within the Veterans population; this data is timely considering the implementation of the VHA’s Patient Aligned Care Teams (PACT), the patient-centered Medical Home model adopted within the VA system that occurred in 2009. The Medical Home Model attempts to bend the cost curve downward by providing coordination and continuity of care and avoiding unnecessary duplication of services. A major outcome metric in the Medical Home model is to reduce unnecessary use of emergency department and inpatient services. Appropriate primary care management is expected to reduce levels of “ambulatory care sensitive conditions” which are responsive to the coordinated medical management provided in the Medical Home Model. As such, these data provide baseline data to determine if health literacy associated cost outcomes are impacted by the implementation of PACT within the VA system.

These findings also have global implications within and beyond the VA system. Within the VA system, as the VACAA is implemented, health literacy remains a cost issue and should be addressed for veterans who may have health literacy needs. Veterans, under certain circumstances, are now going to have the option to go to non-VA Choice Program providers who may not be as sensitive as the VA providers are to the cost implications of health literacy. Beyond the VA system, these findings have potential implications for observing health literacy and its cost consequences as the ACA is implemented and funded. Though the ACA is designed to give the nation’s population improved access to health care, costs will still be adversely impacted by individuals’ unmet health literacy needs. The proposed ACA’s Accountable Care Organization models for transferring financial risk to providers are a currently a work in progress. The VHA is a laboratory of comprehensive and coordinated integrated medicine. These data findings have policy relevance within and beyond the VA. Health literacy adversely impacts health status and costs even in an integrated system, as shown by this study’s findings. If this is true within a sophisticated integrated healthcare system such as the VHA, with a mature electronic medical records system, and a comprehensive primary care Medical Home model, it can be inferred that the same will be the case for the nation’s healthcare system. Access to insurance under the ACA and the shifting of financial risk to Accountable Care Organizations may not be enough to simultaneously improve health status and lower the overall costs of healthcare.

The recognition of the increased costs within the VHA’s integrated system due to unmet health literacy needs provides the justification for interventions within and beyond the VHA system to reduce costs associated with patients’ unmet health literacy needs. Implications of this work indicate screening, and documenting health literacy at the population-level underlies any effort of monitoring and remediating at the patient- and system-levels. This study suggests that considerable gains might be attained by designing organized, evidence-based interventions that target improving health literacy among veterans, and especially those with key chronic conditions. In a fully-integrated national system, this has significant implications in an era of healthcare reforms focused on patient-centered care and improved care coordination. Administrative datasets derived from electronic medical records can be leveraged to support efforts to identify and monitor the rate of health literacy and its associated impact on health care utilization and costs.

As health literacy continues to gain attention at the national level and cost outcomes become more apparent, mitigating health literacy issues will be critical in reducing costs and other adverse outcomes associated with low health literacy. To date, there are no universal solutions however best practices indicate strategies can be systematically used to improve health literacy for priority populations within specific contexts of care. Improving the usability of health information is a primary means of supporting individuals’ health literacy needs, as well building knowledge to improve individuals’ decision making skills. This is most commonly done with the use of plain language in spoken and written health information. Another key element and innovative concept in improving health literacy as improving the usability and navigation ease of health services, which require some level of effort in advocating health literacy at the organizational level. The approach of assessing and responding to health literacy at the organizational has been an evolution in the focus of health literacy as a responsibility of the individual, to a perspective that holds the organization and system accountable for designing service delivery in a way that meets the health literacy needs of all patient consumers.

### Study limitations

Study limitations should be considered when interpreting our findings. First, we used a regional population based study design which may not be representative of other regions in the United States; however, the use of population based data at the regional level affords us considerable power, with stable estimates. Second, “lost to follow-up” can be an issue in cohort studies; however our study was a population based study of veterans who used care in each of the three follow-up years. Third, cross-sectional studies cannot be used to confirm causality. Finally, the data presented is limited to services documented and paid for by the VA system. Some veterans may have expenses not accounted for in this system, such as Medicare, Medicaid, or from private health insurance. Our analyses could underestimate the total healthcare use and costs among dual users, which could bias our results. This is an unfortunate, yet common limitation, when conducting analyses using VHA data, however these findings warrant further investigation using other data sources, such as non-VHA cost CMS data available from the VA-CMS data merge project and using data in future research from the Fee Basis program. Our cost estimates are derived internally from administrative systems as best estimates for the delivery expense, and would not necessarily be reflective of a market price; however, our purpose was to examine the relative costs of care associated with health literacy levels.

## Conclusions

In summary, patients’ health literacy is known to be an important factor impacting health status and costs in non-integrated systems and remains a significant issue in a comprehensive integrated health care system such as the VHA. In this study, the estimated VA medical and pharmacy cost associated with veterans’ with marginal and inadequate health literacy for a three-year period, using mean values, was approximately $143 million dollars more than those with adequate health literacy. These data suggest 17.2 % (inadequate & marginal) of the population accounts for almost one-quarter (24 %) of VA medical and pharmacy cost for this 3-year period. Meeting the needs of those with marginal and inadequate health literacy could produce potential economic savings of approximately 8 % of total costs for this population in this three-year timeframe. To meet patients’ health literacy needs, health systems such as the VA need to prioritize a comprehensive approach to implementing a combination of strategies including the use of spoken and written plain language materials in tandem with improving patients’ decision making skills, while improving the usability and navigation ease of health services at the organizational level.

These findings warrant further research to understand the mechanisms by which higher costs result in veteran populations with lower health literacy. Additional studies to analyze data after 2009 can also examine the relative impact of implementing a patient centered primary care medical home model (PACT) on the identified mechanisms and costs associated with lower health literacy. Furthermore, these data represent a relative baseline to understanding the impact of health literacy in the era of the VACAA and ACA implementation.

## Abbreviations

VACAA: Access, Choice, and Accountability Act
ACA: Affordable Care Act
CI: Confidence intervals
DSS-NDE: Decision Support System - National Data Extracts
VA: Department of Veterans’ Affairs
DCG: Diagnostic Cost Group
FY: Fiscal year
HCC: Hierarchical Condition Category
PACT: Primary care medical home model
VHA: Veterans Health Administration
MedSAS: VHA Medical SAS
